# Mitochondrial localization of the OAS1 p46 isoform associated with a common single nucleotide polymorphism

**DOI:** 10.1186/1471-2121-15-33

**Published:** 2014-09-09

**Authors:** Karina Hansen Kjær, Jytte Pahus, Mariann Fagernæs Hansen, Jesper Buchhave Poulsen, Erik Ilsø Christensen, Just Justesen, Pia Møller Martensen

**Affiliations:** 1 Department of Molecular Biology and Genetics, Aarhus University, C.F. Møllers Allé 3, 8000 Århus C, Denmark; 2 Department of Biomedicine, Aarhus University, Wilhelm Meyers Allé 3, 8000 Aarhus C, Denmark

**Keywords:** 2*'*-5*'* Oligoadenylate Synthetase (OAS), Single Nucleotide Polymorphism (SNP), Mitochondria, Diabetes

## Abstract

**Background:**

The expression of 2′-5′-Oligoadenylate synthetases (OASs) is induced by type 1 Interferons (IFNs) in response to viral infection. The OAS proteins have a unique ability to produce 2′-5′ Oligoadenylates, which bind and activate the ribonuclease RNase L. The RNase L degrades cellular RNAs which in turn inhibits protein translation and induces apoptosis. Several single nucleotide polymorphisms (SNPs) in the OAS1 gene have been associated with disease. We have investigated the functional effect of two common SNPs in the OAS1 gene. The SNP rs10774671 affects splicing to one of the exons in the OAS1 gene giving rise to differential expression of the OAS1 isoforms, and the SNP rs1131454 (former rs3741981) resides in exon 3 giving rise to OAS1 isoforms with either a Glycine or a Serine at position 162 in the core OAS unit.

**Results:**

We have used three human cell lines with different genotypes in the OAS1 SNP rs10774671, HeLa cells with the AA genotype, HT1080 cells with AG, and Daudi cells with GG. The main OAS1 isoform expressed in Daudi and HT1080 cells was p46, and the main OAS1 isoform expressed in HeLa cells was p42. In addition, low levels of the OAS1 p52 mRNA was detected in HeLa cells and p48 mRNA in Daudi cells, and trace amounts of p44a mRNA were detected in the three cell lines treated with type 1 interferon. We show that the OAS1 p46 isoform was localized in the mitochondria in Daudi cells, whereas the OAS1 isoforms in HeLa cells were primarily localized in cytoplasmic vacuoles/lysosomes. By using recombinantly expressed OAS1 mutant proteins, we found that the OAS1 SNP rs1131454 (former rs3741981) did not affect the enzymatic OAS1 activity.

**Conclusions:**

The SNP rs10774671 determines differential expression of the OAS1 isoforms. In Daudi and HT1080 cells the p46 isoform is the most abundantly expressed isoform associated with the G allele, whereas in HeLa cells the most abundantly expressed isoform is p42 associated with the A allele. The SNP rs1131454 (former rs3741981) does not interfere with OAS1 enzyme activity. The OAS1 p46 isoform localizes to the mitochondria, therefore a full 2-5A system can now be found in the mitochondria.

## Background

**Figure 1:**
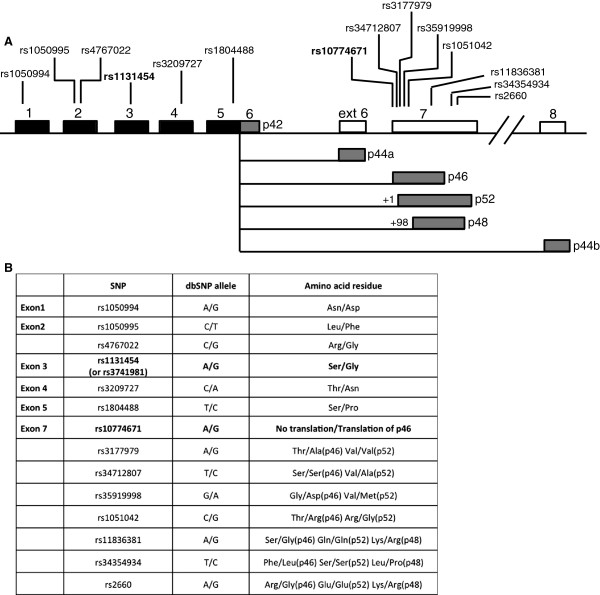
**The human OAS1 splice variants and single nucelotide polymorphisms. A**. Schematic view of the human OAS1 gene and the position of known single nucleotide polymorphisms (SNPs) in the exons. All mature OAS1 mRNAs share the same core unit, exon 1-5 (black boxes), but contain different 3′ exons resulting from alternative splicing (grey boxes). The p42 mRNA variant results from no splicing of exon 5. **B**. The consequence of some of the OAS1 SNPs for the encoded proteins. The SNPs discussed in this paper are marked in bold.

The interferon (IFN) system is activated in response to viral infection and plays an important role in the host defence system. Type I IFNs induce proteins with antiviral activity such as double-stranded RNA-activated protein kinase (PKR), 2′,5′-Oligoadenylate synthetases (OASs), RNase L, and the Mx protein GTPases [1,2]. Our focus here is the OAS family of enzymes, which catalyses the synthesis of oligoadenylates of the general structure ppp(A2′p)*n*A, commonly abbreviated 2-5A. RNase L, a latent endoribonuclease, becomes activated upon binding of 2-5As, and when activated it catalyses the degradation of both viral and cellular RNAs, including ribosomal RNA [3]. This antiviral response results in inhibition of protein synthesis and in some cases it causes apoptosis. Three genes encoding enzymatic active OAS enzymes have been identified in human cells by immunoblotting analyses and characterization of cDNA and genomic clones, and they are designated OAS1, OAS2, and OAS3 [4,5]. The human OAS1 gene encodes at least six isoforms with different theoretical molecular weights, p42, p44a, p44b, p46, p48 and p52 (Figure 1A). The OAS1 isoforms are splice variants and contain the same five core exons but differ in their 3′ exons. The OAS1 isoforms p42 and p46, the OAS2 isoforms p69 and p71 deriving from alternative splicing of the OAS2 gene, and the OAS3 isoform p100 have been detected at the protein level in human cells [6,7], whereas the p52, p48, p44a and p44b OAS1 isoforms have only been detected at the mRNA level in cells treated with IFN [4,8-10].

It has previously been demonstrated that the cellular localization of the OAS proteins differs. It has been shown by electron microscopy that the human OAS2 p69 isoform is localized to the membranes of the nuclear envelope and rough endoplasmic reticulum [11]. The OAS3 p100 isoform is localized to the cytoplasm [12], whereas the OAS1 p42 isoform was found mainly in the cytoplasmic fraction and the p46 isoform mainly in the microsomal fraction of cells [6].

The expression of the OAS1 isoforms are cell-type specific [6,7,13], and the differential expression patterns of some of the OAS1 isoforms can be related to a single-nucleotide polymorphism (SNP) rs10774671 situated in the splice acceptor site at the 5′ end of exon 7 in the OAS1 gene (Figure 1) [9]. The two alleles have been named A and G, since one has a splice acceptor site in exon 7 with an A**A**G sequence, and the other has a splice acceptor site with an A**G**G sequence. The AGG splice acceptor site (G allele) results in mRNAs encoding p46, and the AAG splice acceptor site results in an mRNA encoding p52, since the splice site between exon 5 and exon 7 has shifted one nucleotide downstream. The expression of p48 results from splicing 98 nucleotides downstream from the exon 7 start site, and the expression of p42 derives from mRNA linking exon 5 and exon 6 without splicing.

The rs10774671 SNP has been associated with two autoimmune diseases, Type 1 diabetes and mutiple sclerosis. In Type 1 diabetes, it was found that affected persons were predominantly homozygous for the G allele [14], and this association was confirmed by others [15]. These authors further concluded that the rs10774671 SNP was associated with rs1131454 (former rs3741981) as a haplotype in Type 1 diabetics. The rs1131454 SNP (former rs3741981) is an A or G positioned in exon 3 resulting in an OAS1 protein with either Ser162 or Gly162 in the core domain. The association between these two SNPs and Type 1 diabetes has not been confirmed in a larger study, though others have concluded that there may be a very weak association between the rs1131454 (former rs3741981) and rs10774671 SNPs and Type 1 diabetes in a population of European descents, however these authors stated that this association might also be linked to other SNPs in the OAS1 locus [16,17]. In multiple sclerosis the same haplotype was studied in a Spanish study group [18]. They found that the G allele of rs10774671 and the A allele of rs1131454 (former rs3741981) seemed to increase the risk of having multiple sclerosis, which is the same risk haplotype seen for Type 1 diabetes. In an Irish study, only considering the rs10774671 SNP, the opposite was found, in that the G allele showed to be protective of multiple sclerosis whereas the A allele conferred susceptibility [19]. The AA genotype was associated with disease activity despite IFNβ therapy and earlier breakthrough of multiple sclerosis upon IFNβ therapy, whereas the GG genotype was associated with delayed breakthrough relapses upon IFNβ therapy. Finally, in an Italian study no association was found between rs10774671 and multiple sclerosis [20]. Thus the association with the two OAS1 SNPs, rs10774671 and rs1131454 (former rs3741981), and type 1 diabetes or multiple sclerosis remain elusive.

An association was found with the OAS1 SNP rs10774671 and the response to measles vaccination in an African-American population [21]. The A allele was associated with higher levels of measles neutralizing antibodies in a dose dependent manner with regard to the genotype AA > AG > GG. During rubella vaccinations, the A allele was associated with lower rubella virus specific IL-2 secretion and the G allele with higher IL-2 secretion [22]. It seems as if OAS1 gene polymorphism might also be involved in the mechanism underlying the heterogeneous immune response to rubella and measles vaccinations.

During West Nile Virus (WNV) infections, the AA genotype of rs10774671 resulted in higher virus titers than AG or GG genotypes when tested in an *ex vivo* model of WNV replication in cultured human lymphoid tissue [23]. It was concluded that the rs10774671 is a host genetic risk factor for initial WNV infection in humans. However, this specific association could not be seen in a more recent case-control study, although an association was seen between the SNP rs34137742 positioned in the intron between exon 2 and exon 3 in the OAS1 gene and an increased risk for WNV encephalitis and paralysis [24]. In Hepatitis C virus (HCV) patients it was found that patients with AA in rs10774671 were non-responders to interferon treatment and they had a progressive HCV disease [25]. When overexpressing the individual OAS proteins in human hepatoma Huh7 cells, OAS1 p46 as well as OAS3 p100 could inhibit HCV replication, whereas the OAS1 isoforms p42, p48 and p52 and the OAS2 isoforms p69 and p71 could not [26]. This suggests that expression of p46 caused by the G allele of the rs10774671 SNP is protective of HCV infection, or the A allele could be a risk factor. Overexpression of individual OAS proteins in A549 cells infected with Dengue virus resulted in antiviral effects of the OAS1 p42 and p46 and the OAS3 p100 [27]. The other OAS proteins, OAS1 p44b, p48 and p52 as well as the OAS2 p69 and p71 did not lead to antiviral effects. This suggests that the A allele of the rs10774671 SNP could be a risk factor for Dengue infection. In conclusion, infection of the flaviviruses WNV, Dengue virus and HCV seem to be inhibited by expression of the p46 OAS1 protein derived from the presence of the G allele of the rs10774671 SNP in the OAS1 gene.

Total OAS enzyme activity analysis of peripheral blood lymphocytes from 147 individuals revealed that persons with the A allele in rs10774671 had a lower total OAS enzymatic activity compared with persons with the G allele [9]. The OAS activity decreased in a dose dependent manner, the genotype GG > GA > AA. This could be due to differences in activity of the OAS1 isoforms p52 encoded by the A allele, p46 encoded by the G allele, or differential levels of p48 encoded by either allele. No evidence has yet been presented to support this, as the levels of p46, p48 and p52 OAS1 proteins in these cells were not analysed. The difference in total OAS activity in the leukocytes observed could also be a result of other SNPs in the OAS locus, which could be linked as haplotypes to the A and G alleles of the SNP rs10774671.

In order to clarify some of the consequences of two of the important SNPs (rs10774671 and rs1131454 (former rs3741981)) in the OAS1 gene, we have determined the mRNA expression profiles of the OAS1, OAS2 and OAS3 genes in three different cell lines with different OAS1 genotypes at the rs10774671 position. Furthermore, we have analysed the OAS protein expression patterns of these three cell lines and determined the total OAS enzyme activity after interferon treatment. We have determined the functional effects of rs1131454 (former rs3741981), which results in two isoforms of the OAS1 proteins by analysing two mutants of an OAS1 core protein, one containing a Serine and the other a Glycine at position 162 in the primary structure. Finally, we present evidence of the specific cellular localization of the OAS1 isoform p46 to the mitochondria, which differs from p42.

## Results

In order to address potential influences of the rs10774671 polymorphism (Figure 1) on the OAS1 gene expression and enzyme activities relating to the A and G alleles, we set out to identify the OAS mRNA expression profiles and the OAS enzyme activities in three human cell lines, distinguished by their OAS1 GG, GA and AA genotypes. Through DNA sequencing of genomic DNA retrieved from the three cell lines we found that HeLa cells are homozygous for the A allele (AA), HT1080 cells are heterozygous (AG) and Daudi cells are homozygous for the G allele (GG) with respect to the rs10774671 polymorphism.

### mRNA expression profiles of the OAS splice variants

**Figure 2:**
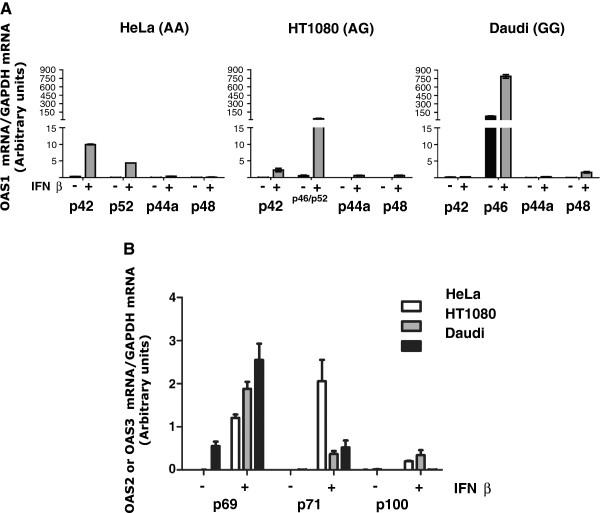
**OAS mRNA expression profiles.** qRT-PCR analyses of the indicated **(A)** OAS1 mRNAs and **(B)** OAS2 and OAS3 mRNAs before (-) and after treatment with IFN-β (+) in HeLa cells (AA genotype), HT1080 cells (AG genotype), and Daudi cells (GG genotype). The mRNA levels were normalized to GAPDH mRNA levels. The experiments were done in triplicates. Error bars are SEM in **(A)**, and SD in **(B)**.

In order to create a full expression profile of the OAS1 splice variants p42, p44a, p44b, p46, p48 and p52, we carried out qRT-PCR analyses to measure the mRNA levels of the different isoforms. Primers were created with a forward primer annealing within the exon 1-5 core region whereas a downstream reverse primer was designed to specifically amplify each splice variant (Figure 1A and Table 1). The p46 and p52 mRNAs could not be distinguished in this qRT-PCR due to only a single nucleotide difference, and several attempts to produce a usable reverse primer failed. We therefore made the assumption that HeLa cells do not express p46 and Daudi cells not p52, as a consequence of the AA/GG genotypes of these cell lines with respect to the rs10774671 polymorphism. HeLa, HT1080 and Daudi cells were treated with IFN-β, or left un-treated as controls for 24 hours. Total RNA was isolated and qRT-PCR analyses were performed using GAPDH for normalization, which did not change during the experimental set-up. The main OAS1 mRNA product found in HeLa cells treated with IFN was the unspliced p42 isoform, with a smaller amount of spliced p52 mRNA and only trace amounts of p48 mRNA detected (Figure 2A). In the heterozygous cell line HT1080 treated with IFN, there was predominantly the spliced p46/52 mRNAs and less of the p42 mRNA. The main OAS1 mRNA in Daudi cells was the spliced p46, and this mRNA was present in control cells at a considerable amount, and the level increased after interferon treatment. Of the other OAS1 isoforms only very lowinducible amounts of p44a and p48 mRNAs could be detected in the three cell lines and we were unable to detect p44b mRNA in any of the cell lines. All qRT-PCR dissociation curves indicated single PCR products. This was confirmed by agarose gel electrophoresis of all the qRT-PCR samples, revealing the amplification of the expected band size for each OAS1 mRNA (data not shown).We then determined mRNA expression profiles of the OAS2 (p69 and p71) and OAS3 mRNAs (p100) in the three cell lines (Figure 2B). The OAS2 p69 and p71 mRNAs were found to increase after interferon treatment in the three cell lines, whereas the OAS3 p100 mRNA was induced in HeLa and HT1080 cells and was barely detectable in Daudi cells. Daudi and HT1080 cells primarily expressed the OAS2 p69 mRNA, whereas HeLa cells expressed both the p71 and p69 mRNAs. Daudi cells also expressed p69 mRNA in untreated cells.

**Table 1:** Primers for quantitative reverse transcriptase PCR

**Target mRNA**	**Forward primer**	**Reverse primer**
**OAS1 p42**	5*'*-GGTCTTGGAATTAGTCATAAACTACCA-3*'*	5*'*-ATGAATGGCAGGGAGGAAG-3*'*
**OAS1 p42/p46**	5*'*-GAGAAGGCAGCTCACGAAAC-3*'*	5*'*-CGTCTGCACTGTTGCTTTCAGCC-3*'*
**OAS1 p48**	5*'*-GGTCTTGGAATTAGTCATAAACTACCA-3*'*	5*'*-GAGTGTGCTGGGTCAGCAGAATCCAG-3*'*
**OAS1 p44a**	5*'*-GGTCTTGGAATTAGTCATAAACTACCA-3*'*	5*'*-AGTGACATGAGCACTGGCTTT-3*'*
**GAPDH**	5*'*-GGTCGGAGTCAACGGATTT-3*'*	5*'*-CCAGCATCGCCCCACTTG-3*'*
**OAS2 p69**	5*'*-GGAAGTTTCTACTGAGCCAGTTGCAG-3*'*	5*'*-GACAACGCCTCCTTTAGATGAC-3*'*
**OAS2 p71**	5*'*-GGAAGTTTCTACTGAGCCAGTTGCAG-3*'*	5*'*-GGTGTCTGCATTGTCGGCACTTTCCAAG-3*'*
**OAS3 p100**	5*'*-GGGTGGCTGTGATTCTGAACTTG-3*'*	5*'*-CCTGTAGGCACACCTGGTGGTACCAGTG-3′

### Protein expression profiles of the OAS isoforms and total OAS enzyme activity

**Figure 3:**
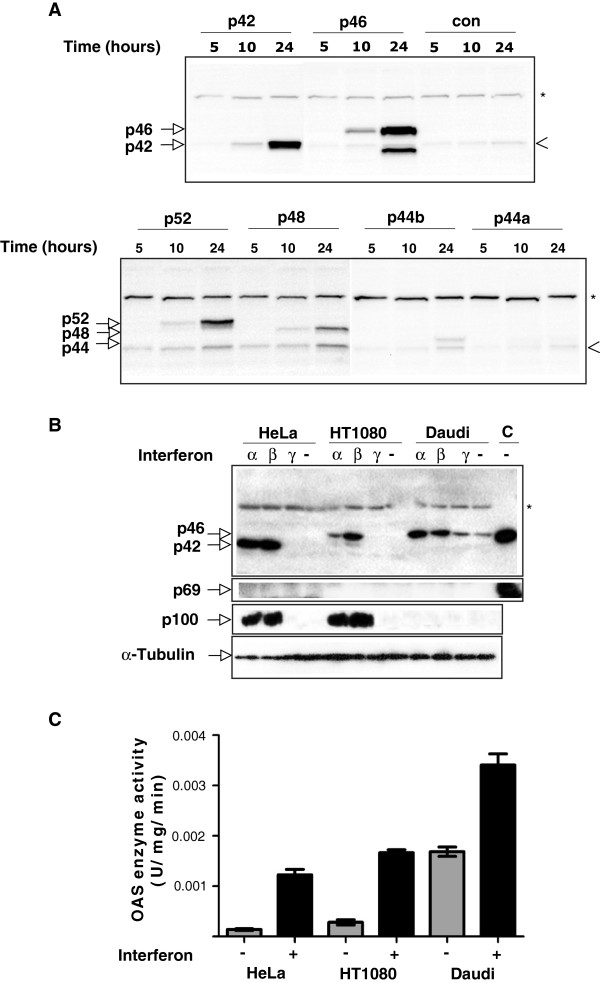
**Cell line-specific OAS protein expression and enzyme activity. A**. HeLa cells were transfected with plasmid constructs encoding the indicated OAS1 variants or the empty control vector. Cells were harvested and lysed at the indicated time after transfection and subjected to immunoblot analysis using the OAS1 specific antibody. The positions of the recombinantly expressed OAS1 isoforms are indicated. The position of the endogenously expressed p42 is indicated by an arrow-head. **B**. Immunoblot analysis of human OAS proteins in HeLa (AA genotype), HT1080 (AG genotype), and Daudi (GG genotype) cells. Cells were either treated with IFN-α, -β and –γ or left untreated (-) for 24 hours, as indicated. The OAS1 proteins were detected by the monoclonal anti-OAS1 antibody, and the OAS2 and the OAS3 proteins with anti-OAS2 and anti-OAS3 antibodies, respectively. C: Protein extracts from HeLa cells transfected with constructs expressing either p46 or p69. α-tubulin was employed as loading control. Unspecific bands recognized by the antibodies are indicated by *. **C**. Total OAS enzyme activity of HeLa, HT1080 and Daudi cell lysates treated with IFN-β (+) or left untreated (-). Enzyme activities were determined using ATP as substrate. The reaction products were visualized by Mono Q chromatography. Peaks were integrated and used to calculate the specific enzyme activities using the total protein concentration in each cell lysate. Results are an average of three independent experiments. Error bars represent SD.

We then carried out immunoblotting analysis of protein extracts from the three cell lines using different anti-OAS antibodies in order to compare the protein levels (Figure 3). Initially, the OAS1 isoforms were analysed by an anti-OAS1 antibody targeting all OAS1 variants, and the ability of the anti-OAS1 antibody to recognize all the OAS1 isoforms was verified by expression of the individual OAS1 isoforms in HeLa cells after transient transfection (Figure 3A). In the cell lines HeLa, HT1080 and Daudi, only the OAS1 isoforms p42 and p46 could be detected (Figure 3B). An IFN-α and IFN-β inducible expression of p42 was seen in HeLa cells, whereas Daudi cells had an IFN-α and IFN-β inducible expression of p46. Daudi cells expressed the p46 protein constitutively, whereas p42 could only be detected after IFN-α and IFN-β treatment of the HeLa cells. In the heterozygous HT1080 cells, the relative levels of expressed protein, i.e. p42 or p46 following IFN-α and IFN-β treatment were in favour of the p46 isoform with the level of p42 being very low. No other OAS1 isoforms were detected in the immunoblotting analysis, most likely due to low levels of protein expression, and the protein expression pattern was found to correlate with the qRT-PCR analyses. We would have expected an expression of p52 in HT1080 and HeLa cells due to the presence of the A allele of the rs10774671 in these cell lines, however the p42 isoform mRNA unspliced between exon 5 and exon 7 prevailed in HeLa cells, and the allele expressing the spliced p46 isoform prevailed in the HT1080 cells.Although a recombinantly expressed OAS2 p69 isoform could be detected in transfected HeLa cells, none of the OAS2 isoforms were detected in the cell lines, even though their mRNAs were present in high amounts (Figure 3B). We tried several commercially available OAS2 antibodies, however none of these detected endogenous expression of the OAS2 proteins. When using an OAS specific peptide antibody recognizing all OAS isoforms, we observed that the OAS2 proteins were indeed expressed in these cell lines in accordance with the OAS2 mRNA levels (data not shown). And finally, the OAS3 isoform p100 was expressed in IFN-α and IFN-β treated HeLa and HT1080 cells, but not in Daudi cells, also in accordance with the mRNA levels. None of the OAS isoforms were induced by IFN-γ in any of the three cell lines.

Total OAS enzyme activity assays were carried out on crude cell extracts from the three cell lines, untreated and treated with IFNβ (Figure 3C). We found that HeLa and HT1080 cells had equal inducible OAS enzyme activities. This OAS activity originated from the OAS1 isoforms p42 in HeLa and mainly p46 in HT1080, together with the OAS2 isoforms p69 and p71, and the OAS3 isoform p100. Daudi cells had a high constitutive OAS activity, which could be increased by IFNβ treatment. The OAS enzyme activity in Daudi cells originated from the OAS1 p46 isoform and the OAS2 p69 and p71 isoforms. The constitutive expression of OAS1 and OAS2 in the Daudi cell line could be due to it originating from a B lymphoblast, since B-cell lymphocytes are known to constitutively express various cytokines [28].

### Enzyme activities of OAS1 mutants associated with the rs1131454 (former rs3741981) polymorphism

**Figure 4:**
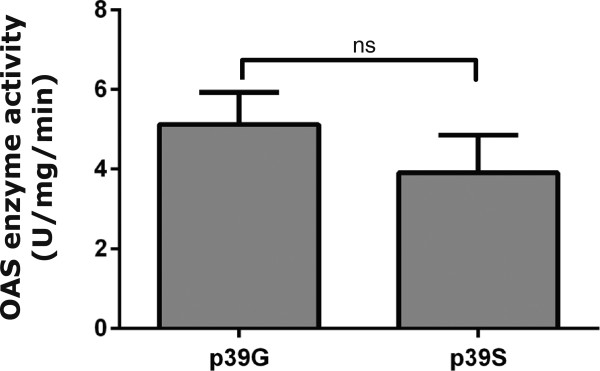
**Specific enzyme activity of p39G and p39S truncated OAS1 variants.** The indicated proteins were purified after expression in *E.coli.* Purified proteins were analysed for OAS activity with ATP as substrate. The reaction products were visualized by Mono Q chromatography. Peaks were integrated and used to calculate specific enzyme activities. Results are an average of three independent assays. Error bars represent SEM.

We next wanted to analyse if the SNP rs1131454 (former rs3741981) (Gly or Ser at position 162 in OAS1) influences the OAS1 enzymatic activity considering its position in the OAS1 core unit. Thus we measured the specific OAS enzyme activities of two truncated OAS1 proteins i.e. p39G/p39S distinguished by only a single amino acid mutation (Gly/Ser) at position 162 giving rise to proteins of ~39 kDa (p39). The specific activities of the p39G and p39S variants were found not to differ (Figure 4). We therefore concluded that the specific activity of the OAS1 isoforms with Gly162 or Ser162 deriving from the rs1131454 (former rs3741981) SNP have indistinguishable specific enzyme activities.

### Cellular localization of the OAS1 p46 and p42 isoforms

**Figure 5:**
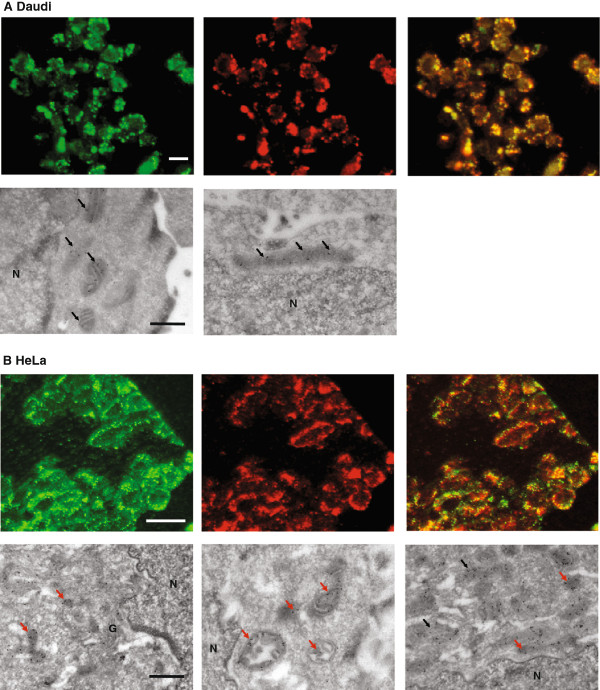
**Cellular localization of endogenous OAS1 p42 in HeLa and p46 in Daudi cells. (A)** Daudi cells and **(B)** HeLa cells were treated with IFN-β for 24 hours. The cells were fixed and prepared for immunofluorescence microscopy (upper panels), which was carried out with the anti-OAS1 antibody and a FITC-conjugated secondary antibody (green). p46 OAS1 was detected in Daudi cells and mainly p42 OAS1 in HeLa cells. Red fluorescence shows the localization of the mitochondrial specific protein VDAC1. Yellow fluorescence indicates the degree of co-localization between the OAS1 proteins and the mitochondrial protein in the merged pictures. Electron microscopy (lower panels) shows localization of the p46 OAS1 protein in Daudi cells and mainly p42 in HeLa cells. Gold particle labeling demonstrates the localization of the OAS1 proteins. Black arrows indicate OAS1 proteins in the mitochondria and red arrows OAS1 proteins in vacuoles/lysosomes in the cytoplasm. The white bars are 10 μm and the black bars 0.5 μm. N: the nucleus. G: Golgi apparatus.

Previous analyses of the localization of the OAS1 isoforms have been conducted using either highly expressed recombinant proteins or using antibodies recognizing all OAS isoforms. Using HeLa and Daudi cells expressing mainly p42 or p46 combined with the very specific OAS1 antibody, we were able to distinguish the cellular localization of these two OAS1 isoforms.HeLa and Daudi cells were stimulated with IFN-β for 24 hours and fixed cells were then subjected to immunofluorescence cytochemistry and electron microscopy using the anti-OAS1 antibody (Figure 5). The fixed cell pellets were cryosectioned, and this allow for analyses of endogenous proteins without prior permeabilisation and a good resolution of the immunofluoresence cytochemistry. As a mitochondrial tracker in these immunofluoresence analyses, we used an antibody against the mitochondrial protein VDAC1. The results obtained from Daudi cells showed that the OAS1 p46 isoform, which is the main OAS1 isoform present in these cells together with very low amounts of p48 detected on the mRNA level (Figure 5A, green), co-localized with the mitochondrial protein (red), and this was clearly seen in the merged picture (yellow). The localisation of p46 to the mitochondria was also supported by electron microscopy (Figure 5A, bottom images), showing concentrated staining of p46 in the mitochondria. The cellular localization of the OAS1 isoforms in HeLa cells is different. The OAS1 isoforms distributed throughout the cytoplasm (Figure 5B, green), which does not perfectly co-localize with the VDAC1 mitochondrial protein (Figure 5B, red), seen in the merged picture as yellow stain. This finding is supported by the electron microscopy, showing that the HeLa OAS1 isoforms associated with organelle structures resembling vacuoles/lysosomes in the cytoplasm as well as with the mitochondria (Figure 5B, bottom images). The main OAS1 isoform present in HeLa cells was p42 together with low amounts of p52 detected on the mRNA level.

**Figure 6:**
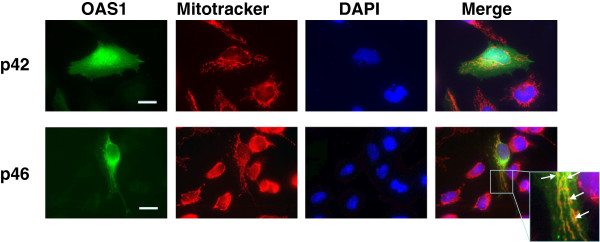
**Cellular localization of overexpressed OAS1 p42 and p46 isoforms in HeLa cells.** HeLa cells were transfected with plasmids encoding OAS1 p42 or p46, as indicated. Prior to fixation the cells were treated with Mitotracker (red). The cells were incubated with the OAS1 primary antibody and a FITC-conjugated secondary antibody (green). The nuclei were stained with DAPI (blue). Yellow fluorescence indicates the degree of co-localization between the OAS1 p42 or p46 proteins and the mitotracker in the merged pictures. The frame in the merged picture indicates the magnified area. Arrows indicate some of the positions of co-localization. The white bars are 10 μm.

In order to establish if the mitochondrial localization of p46 was cell type specific for Daudi cells, we overexpressed OAS1 p46 and p42 in HeLa cells after transient transfections (Figure 6). Also in HeLa cells, p46 is localised to the mitochondria seen as a co-localization with the Mitotracker. Overexpressed p42 distributed evenly in the HeLa cells, as we have seen previously with overexpressed His-tagged OAS1 p42 protein [29]. Since the transfected HeLa cells were not treated with interferon, no endogenous OAS1 proteins could be detected in the un-transfected cells.

We therefore concluded that the OAS1 p46 isoform localized in the mitochondria, whereas the OAS1 p42 isoform mainly localized in vacuoles/lysosomes within the cytoplasm as well as in the mitochondria.

## Discussion

Several theories have been proposed regarding the association of SNPs in the OAS1 gene with different diseases, but no direct correlation between the SNPs and the expression pattern and specific enzymatic activities has been shown up till now. The OAS1 mRNAs can be differentially spliced according to a polymorphism at the splice acceptor site of exon 7 (rs10774671), and it has been observed that lymphocytes from individuals with the G allele have a higher total OAS enzymatic activity than individuals with the A allele [9].

Bonnevie-Nielsen *et al*. [9] showed in leukocytes that the A and G alleles of the rs10774671 SNP could be associated with the presence of either the p48 and p52 or the p46 OAS1 mRNAs, respectively. However, the individual levels of OAS1 proteins were not investigated, and the level of p42 mRNA unspliced between exon 5 and exon 7 was not analysed either. We have now demonstrated that the A allele present in HeLa cells primarily leads to expression of the unspliced p42 mRNA and only secondary to the spliced p52 mRNA, whereas the G allele present in Daudi cells leads to expression of the spliced p46 mRNA and to a lower extent of p48 mRNA. Only the p42 and p46 isoforms could be detected by immunoblotting analyses, whereas p44a, p48 and p52 mRNAs were only detected by qRT-PCR. The finding of p42 and p46 as the main OAS1 proteins expressed is also supported by earlier immunoblotting analyses [30].

Very recently, the rs10774671 polymorphism was analysed in human bronchial epithelial cells [10]. In bronchial cells with the GG genotype only the p46 isoform was detected, whereas in the cells with the AA genotype the expression of the unspliced p42 isoform prevailed the expression of the spliced isoforms p44a, p48, and p52, as the latter three were only detected at the mRNA level. Also in HT1080 cells, previous results showed that the endogenous level of the OAS1 p48 isoform was very low [8]. Accordingly, the A allele most likely prompts primarily to the production of the unspliced p42 isoform, and the G allele the production of the spliced p46 isoform. However, it can not be excluded that p48 and p52 might be expressed at higher levels in leukocytes harbouring the A allele together with p42, since splicing is cell type-specific.

Although debated, it has been suggested that the SNPs rs10774671 and rs1131454 (former rs3741981) may be linked as a haplotype [15]. We have analysed the functional effect of the rs1131454 (former rs3741981) SNP, and found that the p39G truncated OAS1 variant exhibited the same specific OAS enzyme activity as the p39S variant. Consequently, the increased OAS enzyme activities in leukocytes with the G allele is unlikely to be caused by a higher specific enzyme activity of Gly162 containing OAS1 variants compared with the Ser162 containing OAS1 variants. The contribution of the the OAS2 and OAS3 proteins to the total OAS activity in the three cell lines investigated here is not clear, and further experiments are required to address this issue.

Importantly, however, we have shown that the OAS1 isoforms in HeLa and Daudi cells harbour a differential cellular localization. The main OAS1 isoform p46 expressed in Daudi cells was found to be localized in the mitochondria and no OAS1 was found in the cytoplasm. In HeLa cells, the main OAS1 isoform p42 was localized in cytosolic compartments resembling vacuoles/lysosomes as well as in the mitochondria. When the OAS1 p46 isoform was overexpressed in HeLa cells, it also localized in the mitochondria ruling out the possibility of a cell type specific localization of p46 in Daudi cells. This supports earlier findings that p42 and p46 are in the cytoplasmic as well as in microsomal fractions, p42 mostly in the cytoplasm and p46 mainly in association with the microsomal fraction [6], which probably contained the mitochondria.

The expression of the OAS1 isoform p46 encoded by the presence of the G allele leads to: (1) High OAS enzyme activity in leukocytes from Type 1 diabetics, (2) Low response to measles vaccination and higher virus-specific IL-2 secretion during rubella vaccination, and (3) Inhibition of WNV, HCV and Dengue virus replication. Additionally it has been documented that the G allele hence p46 seems to predominate in skin fibroblasts derived from Alzheimer disease patients [31]. The difference in cellular localization between the p42 and p46 isoforms identified here could potentially explain part of these associations. The presence of p46 in the mitochondria may lead to longer half-life of the protein, and thus sustained OAS activity in the cells. The shift in OAS enzyme activities towards mitochondria may improve conditions for apoptosis, since mitochondria is a key player in the intrinsic apoptotic pathway [32]. Both of these hypotheses are speculative at the moment.

Although our goal was to unravel the putative functionality of the rs10774671 and rs1131454 (former rs3741981) OAS1 SNPs, the identification of the p46 OAS1 isoform in mitochondria adds to the understanding of the 2-5A system. We have previously found a conserved CaaX motif in a number of OAS1 isoforms [33], which are conserved in several species [34]. The CaaX motif is known to be important for geranylation or farnesylation of protein targets, and is believed to functionally direct these to cellular membranes. We therefore suggest that the localization of the different OAS1 isoforms could differ depending on the presence of this CaaX motif in the C-terminus. In humans, only the p46 isoform holds this C-terminal CaaX motif, while the other OAS1 isoforms including p42 do not. Further experiments are required to investigate this hypothesis.

**Figure 7:**
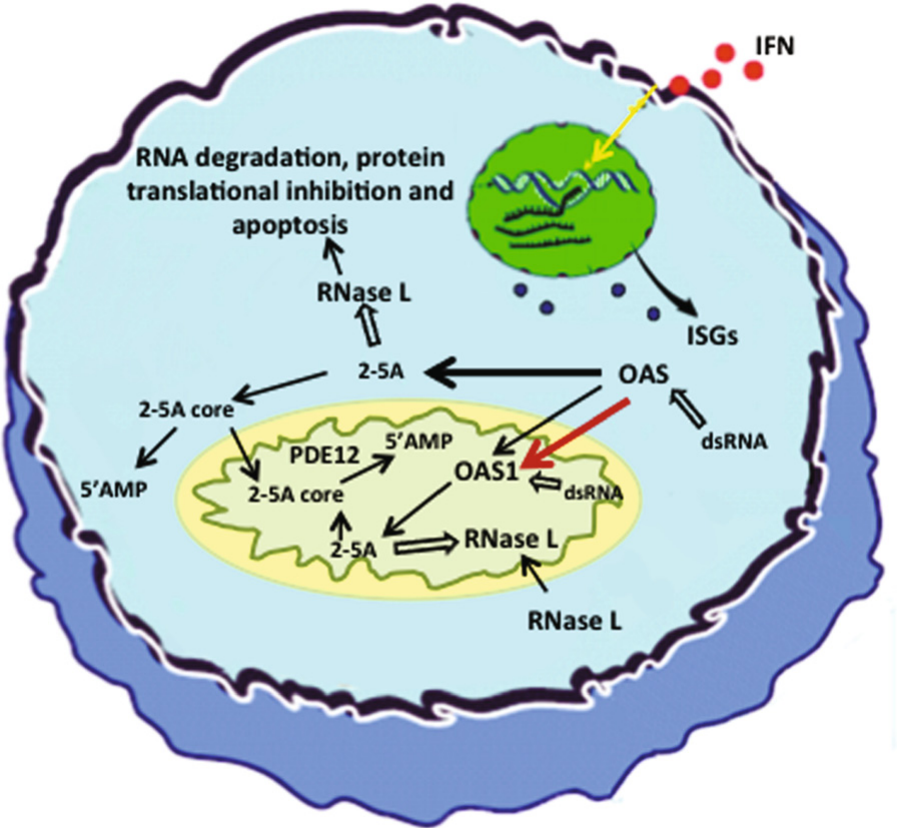
**Model of the 2-5A system.** OAS and RNase L, two of the enzymes constituting the 2-5A system, localizes to the cytoplasm as well as to the mitochondria rendering a full 2-5A system present in both the cytoplasm and the mitochondria (light green/ivory). Individuals carrying the G allele of polymorphism rs10774671 generally have a higher amount of OAS protein in the mitochondria represented by the OAS1 p46 isoform (red arrows), whereas individuals harbouring the A allele in general have higher levels of OAS in the cytoplasm (black arrows). The 2-5As synthesized in the cytoplasm, activate cytosolic RNaseL and are themselves regulated by an unknown 5′-phosphatase. The resulting products, 2-5A core molecules are transported to the mitochondrial matrix and degraded by a 2′ phosphodiesterase (known as PDE12), or alternatively, degraded by an unknown 2′phosphodiesterase located in the cytosol. 2-5As are also synthesized in the mitochondria leading to activation of mitochondrial RNaseL. The 2-5As are here prone to direct degradation by PDE12, or alternatively trimmed by a phosphatase before degradation by PDE12. The cell is not drawn to scale.

We have previously shown that the 2′-phosphodiesterase PDE12, a nuclease degrading 2-5As is also localized to the mitochondria or more specifically to the mitochondrial matrix [29]. This mitochondrial localization was found puzzling with regard to the 2-5A system, since no OAS enzymes at that time had been found in mitochondria. Accordingly, if most 2-5As were produced and active in the cytoplasmic compartment, the mitochondrial inner membrane would logically pose a hydrophobic barrier hindering the PDE12 from accessing its 2-5A substrates. Previously, an activated RNase L was also found in the mitochondria although a broad cellular distribution was observed for this enzyme [35-37]. It is therefore now possible to put forward a model with a complete 2-5A system in the mitochondria (Figure 7). Since the PDE12 share similarities with other RNA deadenylases and was found to degrade 3′-5′ RNA linkages also, it was suggested that it had a role in mitochondrial RNA turnover. Our results and results from others indicated that the PDE12 was indeed regulating mitochondrial mRNAs [29,38]. In addition, it has also been suggested that mitochondrial RNase L might influence mitochondrial mRNA turnover and induce apoptosis, when cells are stimulated by IFNα [39]. If this is the case, the p46 isoform might contribute to the regulation of mitochondrial RNA turnover as an activator of RNase L. Hence, the p46 isoform might be involved in the pro-apoptotic stimulation of RNase L in mitochondria.

## Conclusions

We have found that the SNP rs10774671 in the OAS1 gene mainly leads to expression of either the p46 isoform or the p42 isoform. In the SNP associated with diabetes, the expression of the OAS1 isoform p46 prevails, and we have seen that this isoform is localized to the mitochondria. Our data support a model that in certain conditions the mitochondria can harbour a full 2-5A system, which might contribute to interferon stimulated apoptosis.

## Methods

### Cell lines

HeLa (ATTC: CCL-2) and HT1080 (ATCC: CCL-121) cells were cultured as adherent cells at 37°C with 5% CO2 in Dulbecco’s modified Eagle’s medium containing 10% fetal bovine serum and 1% Penicillin/Streptomycin. Daudi cells (ATCC: CCL-213) were cultured as suspension cultures using similar conditions in Roswell Park Memorial Institute medium with 5% fetal bovine serum and 1% Penicillin/Streptomycin. The cells were grown to 80% confluence or 10^6^ cells/ml and treated with 500 units/mL of IFN-α, 500 units/mL of IFN-β, 100 units/mL of IFN-γ or left untreated as controls for 40 hours. Adherent cells were harvested with a cell scraper, pelleted at 230 ×g for 10 minutes at 4°C and washed once in PBS. The Daudi cells were harvested by centrifugation and washed once. Transfections of HeLa cells with either OAS1 cDNAs or OAS2 p69 cDNA under the control of the CMV promotor and control plasmids were performed with either TurboFect Transfection Reagent (Fermentas) following the manufactures protocol or using the polyethylenimine (PEI) method [40] for the immunofluoresence cytochemistry studies. In brief, linear PEI (25,000 Da) was used and complexes of DNA:PEI at ratios 1:8 (w/w) were allowed to form and added to the cells. Cells were incubated for 24-25 hours after transfection, and during this time the growth medium was changed five hours after addition of the DNA:PEI complexes.

### Purification and sequencing of genomic DNA

Untreated cell pellets were washed once in nuclei lysis buffer (0.1 M Tris EDTA, pH 8.0; 0.4 M NaCl; 2 mM EDTA). The pellets were then resuspended in the same nuclei lysis buffer, pre-added 2 mg/ml Proteinase K and 8% SDS, and incubated over night at 45°C. The cell lysates were added NaCl to a final concentration of 4 M, and the tubes vortexed for 15 sec. Samples were then centrifuged at 845 ×g for 15 min. The supernatants were centrifuged repeatedly until samples were clear of salt, at least 3 times. Cleared supernatants were then added 1/10 volume of 3 M sodium acetate (pH 5.2) and 3 volumes of cold isopropanol and gently shaken. The precipitated DNA was resuspended in 70% ethanol, and tubes were placed on inverting racks for 2 hours. The DNA threads were then isolated and centrifuged for 30 minutes at 18,400 ×g. DNA pellets were dried, and resuspended in ddH_2_0, left at 37°C overnight, and stored at -20°C. The DNA of interest covering the rs10774671 SNP was PCR amplified using the primers; Fw: 5′-GCTAACAAGTGGTGGAAA-3′, Rv: 5′-AAATGAAGGAACTGGTCC-3′, and the amplified product gel-extracted with the DNA and gel band purification kit (GE Healthcare). DNA sequencing was performed with the BigDye Terminator Kit (Applied Biosystems) using the same primers as in the amplification reaction.

### mRNA purification and first strand synthesis

Total mRNA was purified using the QuickPrep Micro mRNA purification kit from Pharmacia. cDNAs were synthesized using the SuperScriptTMII Reverse Transcriptase kit (Invitrogen) with 100 ng of mRNA and 2 pmol of Oligo(dT)_18_ primer.

### Quantitative reverse transcriptase PCR analyses

Quantitative reverse transcriptase (qRT)-PCR reactions were carried out using the SYBR Green qPCR Supermix universal kit (Invitrogen) on a Stratagene Mx3005p. The utilized primers are listed in Table 1. The reaction conditions were optimized with regard to annealing temperature and primer concentration, and the reaction mixes were as follows: 1 × Platinum SYBR Green qPCR SuperMix-UDG (Invitrogen), 0.2% ROX Reference Dye, 250 nM of each primer and 1 μl of template cDNA in total volumes of 20 μl. The results were obtained with the Mx3005P System (Stratagene), running a thermal program of 95°C for 10 min and then 40 cycles of [95°C for 10 s; 55°C for 20 s (at the end of which the SYBR green fluorescence was measured); and 72°C for 20 s]. After this, the samples were heated to 95°C for 1 min and lastly cooled to 55°C, and dissociation curves were obtained by measuring the decay of SYBR green fluorescence when heating the samples from 55 to 95°C. Accurate PCR product band sizes were verified by standard agarose gel electrophoresis, and melting curve analysis of all four PCR products displayed single melting peaks. DNA fragments were in turn excised from the gel, purified as described above and identified by sequencing. All obtained signal levels were normalized to the ROX reference dye levels for correction of errors due to differences in plastic ware transparency and reflectivity or aliquoting errors. Overall threshold fluorescence levels were determined with the default adaptive baseline method of the MxPro Software, and the resulting *C*_t_ values were converted to quantities using the comparative *C*_t_ method. This calculation incorporated the efficiencies of the reaction with each primer set, which were determined. Efficiency was measured using a standard curve generated by serial dilutions of the cDNA. The PCR efficiency (E) was calculated by the formula: E = 10 (1/-slope) - 1, and ranged from 90–110% in the different assays (a slope of -3.32 is equivalent to 100% PCR efficiency) [41]. Finally, each result was normalized to the GAPDH housekeeping gene, which did not change levels during the experiment.

### Immunoblotting analysis

Cells treated with interferons or left untreated as controls were lysed in Radioimmunoprecipitation assay buffer (150 mM NaCl, 10 mM Tris, pH 7.2, 0.1% SDS, 1% Triton X-100, 1% Deoxycholate, 5 mM EDTA). Equal amounts of total protein extracts were subjected to 10% SDS-PAGE and transferred to PVDF membranes. Proteins were detected using the following antibodies: A mouse monoclonal human anti-OAS1 antibody (a kind gift from Illumigen Bioscience Inc., Seattle, USA), a rabbit peptide OAS antibody (raised against the peptide B as described [6], a kind gift from professor Vagn Bonnevie-Nielsen, Vancouver, Canada), anti-OAS2 (abcam), anti-OAS3 (C-15, Santa Cruz), and anti-α-tubulin (Sigma) together with appropriate HRP-conjugated secondary antibodies (donkey anti-goat, Santa Cruz; goat anti-mouse, GE Healthcare; goat anti-rabbit, Dako Denmark). Proteins were visualized using the ECL plus chemiluminescence reagent (GE Healthcare).

### OAS enzyme activity assay

Cells treated with IFN-β and untreated controls were lysed in lysis buffer E (0.5 M CH3COOK, 1% NP-40, 20 mM Tris-HCl, pH 7.8, 1 mM DTT, 0.2 mM EDTA, 5 mM Mg(OAc)_2_ and 10% glycerol). Total protein concentrations were measured with the BCA kit (Pierce). Total OAS activity was measured as described in [29,42] using protein extracts in a total volume of 200 μL with 0.1 mg/ml of Creatine Kinase and 6 mM of Creatine Phosphate.

Specific OAS enzyme activities were determined using 165 ng of the purified His Tagged proteins p39G and p39S (kind gifts from Illumigen Bioscience Inc., Seattle, USA) in the assays. The proteins are human truncated OAS1 isoforms, obtained by recombinant expression in *E. coli* followed by a two-step purification using Ni-NTA and gel filtration chromatography. The p39G and p39S are truncated isoforms of OAS1 p42 (Accession nr. 1207325A) containing only the first 355 amino acid residues with either a Gly or a Ser at amino acid residue position 162.

### Immunofluorescence cytochemistry and electron microscopy

Interferon treated Daudi and HeLa cells were fixed at room temperature in 0.01 M phosphate-buffered saline (PBS), pH 7.4 with 4% formaldehyde for 30 min for immunofluorescence cytochemistry or 2% formaldehyde and 0.1% glutaraldehyde for 10 min for electron microscopy. The cells were washed three times and removed from the support with a rubber policeman in the same buffer, containing 1% gelatine (Merck), pelleted and embedded in 12% gelatine, and finally infiltrated with 2.3 M sucrose in PBS for 30 min, and frozen in liquid nitrogen. For light microscopy, semithin (0.8 μm) cryosections were sectioned at -80°C on a FCS Reichert Ultracut S cryoultramicrotome and preincubated with 0.01 M PBS, 1% BSA or 0.1% milk, 0.05 M glycine, pH 7.4, followed by incubation with primary antibodies (mouse anti-OAS1,1:100 and rabbit anti-VDAC1, 1:10 (Abcam)) for one hour and subsequently by fluorescence conjugated secondary antibodies for one hour at room temperature. The sections were examined in a Zeiss Axioplan fluorescence microscope.

Ultrathin cryosections (70 - 90 nm) were obtained at about -100°C and collected on 200 mesh Ni grids. The sections were incubated overnight with a mouse anti-OAS1 antibody 1:100 prior to 2 h incubation with goat anti-mouse gold (10 nm) (BioCell, Cardiff, UK). All incubations were performed at 4°C. The sections were finally contrasted with methyl cellulose containing 0.3% uranylacetate and studied in a FEI CM100 electron microscope.

For overexpression of OAS1 p42 and p46, HeLa cells were seeded on coverslips and transfected with plasmids encoding p42, p46 or control plasmids using the PEI method (see above). After 24-25 hours, 100 nM of Mitotracker Red CMXRos (Molecular Probes, Invitrogen) was added to the cells for 25 minutes. Cells were then fixed with 4% paraformaldehyde, washed in PBS, permeabilized with 0.05% Triton X-100 for 10 minutes, washed twice with PBS and finally blocked with 3% BSA in PBS-Tween. The cells were then incubated with the mouse anti-OAS1 antibody (1:200) followed by incubation with a FITC-conjugated anti-mouse antibody (1:250, Sigma). The coverslips were mounted with ProLong Gold with DAPI (Molecular Probes, Invitrogen) for visualization of the nuclei. Regular wide field epifluorescence microscopy was performed on a Zeiss Axiovert 200 M Inverted Fluorescence Microscope equipped with a 63× oil-immersion lens (NA 1.4) and an HBO lamp. Images were collected with a Photometric CoolSnapTM HQ camera from Roper Scientific controlled by MetaMorph acquisition software. The images were analysed with ImageJ [43] and Photoshop.

## Abbreviations

OAS: 2′-5′-Oligoadenylate synthetase; 2-5A: 2′-5′-Oligoadenylate; SNP: Single nucleotide polymorphism; RNaseL: Ribonuclease L; GAPDH: Glyceraldehyde 3-phosphate dehydrogenase; PDE12: 2′-Phosphodiesterase; IFN: Interferon; PEI: Polyethylenimine.

## Competing interests

The authors declare that they have no competing interests.

## Authors’ contributions

KHK conceived of the study, carried out most of the experiments, analysed data and wrote the manuscript. JP carried out some of the qRT-PCR studies and enzyme assays. MFH carried out some of the qRT-PCR and the statistical analyses. JBP analysed some of the results and helped to write the manuscript. EIC carried out immunofluoresence and electron microscopy and analysed the data. JJ helped to conceive the study and to write the manuscript. PMM conceived of the study, analysed data and wrote the manuscript. All authors read and approved the final manuscript.
